# Daily decrease of post-operative alpha-fetoprotein by 9% discriminates prognosis of HCC: A multicenter retrospective study

**DOI:** 10.18632/aging.102513

**Published:** 2019-12-12

**Authors:** Pei-Yun Zhou, Chao-Ping Yang, Zheng Tang, Yong Yi, Wei-Ren Liu, Meng-Xin Tian, Jin-Long Huang, Wei Gan, Xi-Fei Jiang, Gao Liu, Han Wang, Chen-Yang Tao, Yuan Fang, Wei-Feng Qu, Cheng Zhou, Ruo-Yu Guan, Bao-Ye Sun, Yu-Fu Zhou, Shu-Shu Song, Zhen-Bin Ding, Yuan-Fei Peng, Zhi Dai, Jian Zhou, Jia Fan, Guo-Zhong Gong, Ying-Hong Shi, Shuang-Jian Qiu

**Affiliations:** 1 Department of Liver Surgery, Liver Cancer Institute, Zhongshan Hospital, Fudan University; Key Laboratory of Carcinogenesis and Cancer Invasion of Ministry of Education, Shanghai, China; 2 Department of Infectious Diseases, Institute of Hepatology, The Second Xiangya Hospital, Central South University, Changsha, China; 3 Institutes of Biomedical Sciences, Fudan University, Shanghai, People's Republic of China

**Keywords:** alpha-fetoprotein, hepatocellular carcinoma, hepatectomy, prognosis

## Abstract

Background: Mixed evidence challenges preoperative alpha-fetoprotein (AFP) as an independent prognostic factor for patients with hepatocellular carcinoma (HCC) after hepatectomy.

Results: Daily post-operative decrease of AFP by 9% as compared to the preoperative level (A09) were selected as the Cut-off. The Kaplan-Meier curve showed that A09 was significantly different for OS (P=0.043) and RFS (P=0.03). A decrease in risk by 54% was observed for OS and 32% for RFS in the at-risk population (A09>9%). A better concordance was observed after adding A09 into TNM and BCLC staging systems. Moreover, a consistent concordance was observed in the internal (FDZS5:0.63; FDZS3:0.608) and external (FDZS5:0.85; FDZS3:0.762) validation cohorts, suggesting its prognostic value in HCC population with elevated AFP.

Conclusions: Decrease in perioperative serum AFP rather than preoperative AFP is an independent prognostic factor for HCC patients after hepatectomy. Cut-off A09 significantly discriminates overall and recurrence-free survival and could be interpret into TNM and BCLC staging systems to improve the stratification power for HCC patients with elevated AFP.

Methods: Kaplan-Meier curve depicted the differences of overall survival (OS) and recurrence-free survival (RFS). Nomogram and concordance were employed to evaluate the superiority of the current staging system.

## INTRODUCTION

Hepatocellular carcinoma (HCC) is one of leading cancer causes of death worldwide [1]. Serological tests such as alpha-fetoprotein (AFP) and des-gamma-carboxy prothrombin (DCP), both cost-effective and expedient, have been investigated over the years for the surveillance and early detection of HCC, but consistent evidence remains insufficient to support their use in HCC prognosis [2, 3]. Hence, more efforts to this end are highly warranted [4, 5]. The most widely used biomarker in HCC, AFP, has been critically challenged for its accuracy. Persistent AFP elevation is a risk factor for HCC development, and AFP is one of the most frequently tested parameters in the diagnosis of HCC [6]. However, AFP levels did not increase detection rate when used in combination with ultrasound (US) [7]. Moreover, appropriate cut-offs may limit its sensitivity and specificity for clinical application, and active hepatitis may act as a confounding factor [7, 8]. With chaotic and mixed data, it is still controversial whether preoperative AFP levels represent an independent prognostic factor in patients undergoing resection for HCC.

Serum AFP level at presentation correlates with tumor size and extent [9]. An observational study showed that serum AFP progressively rose as the tumor grew over 5 cm in diameter [10]. It may also serve as an independent predictor of survival even after adjustment for tumor size and histology [11]. Survival in patients with a serum AFP of greater than 10,000ng/mL at diagnosis was significantly shorter as compared with those with a serum AFP <200 ng/mL (7.6 versus 33.9 percent), suggesting lower AFP levels were associated with well differentiated tumors [11]. It is worthy of note that AFP is a significant influencing factor for delisting liver transplant (LT) candidates with HCC [12] and that identifying HCC candidates at low risk of recurrence seem to be superior to Milan criteria [13], which suggests improved performance by incorporating AFP [14]. A reduction of AFP from >1000 to <500 ng/ml before LT significantly improved outcomes [15]. Moreover, AFP also improved discriminatory ability of some prognostic staging systems [16–18], such as biomarker-combined JIS (BM-JIS) and Chinese University Prognostic Index (CUPI).

While others have failed to find such an association, a propensity score matching analysis indicated that AFP >20 ng/mL was not correlated with clinical outcome in terms of recurrence or survival endpoints following curative hepatectomy for HCC [19]. Another study found that AFP was correlated with short-term recurrence (≤6 months) but not with 2-year recurrence [20]. Pretreatment elevation of AFP resulted in no significant survival difference in locoregional thermal ablation (LTA) and hepatectomy cohort, but AFP-L3 and DCP did in LTA [21]. Serum level of AFP is an independent predictor for mortality of HCV-related HCC [22, 23] but not of HBV-related HCC [24], based on which an extrapolation can be made that active hepatitis may serve as a confounding factor for prognostic prediction by AFP [13, 25].

A decrease of AFP after resection in the HCC patients with a high level may present better prognosis than those with an increase, but accuracy of prediction remains inadequately investigated. It is of clinical significance if a transformer for such a cost-effective marker can be identified to improve its prognostic value for HCC. The present study was aimed to evaluate the significance of perioperative reduction ratio of AFP after surgery on survival and recurrence for HCC patients, which may help extend AFP’s clinical application for prediction and enhance stratification ability cooperating with the current staging system.

## RESULTS

### Demographics and clinical characteristics of the eligible patients

Of 710 eligible patients (145 women and 565 men; ages from 18 to 92 years) in the training cohort, 61 were of A09≤9% (age, mean ±SD of 54.62 ±11.02) and 649 of A09 ≥9% (age, mean ±SD of 51.69 ±11.42). The largest majority (602, 84.8%) had liver cirrhosis, and 554 had a single tumor. The maximum tumor dimension was 4.37±2.84cm (A09≤9%) and 4.89±3.23cm (A09>9%). Most tumors were moderately or well differentiated, and 222 (31.27%) showed microvascular invasion. AFP measured 1954.49±6558.55 (A09≤9%) and 3274.63±7588.34 (A09>9%), and AFPDAY was 0.07±0.02 (A09≤9%) and 0.14±0.06 (A09>9%). Half-life of AFP in A09≤9% group (11.78±20.47 day) was longer than A09>9% group (3.92±0.92 day). Hepatitis B surface antigen (HBsAg) was present in 609 (85.77%) patients, and hepatitis C antibody (HCV) in 6 (0.85%) (Table 1). All patients included were of Child-Pugh A/B, ALBI 2/3 (Supplementary Table 3). Of 164 patients in the internal cohort, 23 (14%) patients were of A09≤9% against 6 (40%) of 15 in the external validation cohort (Data not show).

**Table 1:** Demographic and clinical characteristics of the training cohort.

**Characteristic**	**Variable**	**A09≤9%(n=61)**	**A09>9%(n=649)**
Gender	Female/Male	4/57	141/508
Age	years±SD	54.62±11.02	51.69± 11.42
stratified AFP	40–100/100–400/400–1000/1000~	21/24/7/9	109/172/125/243
HBsAg	-/+	6/55	95/554
HBV DNA	0/3/4/5/6	28/12/12/8/1	344/124/93/75/13
HCV	-/+	61/0	643/6
Ascites	mild/middle/large	48/13/0	573/74/2
HLNE	-/+	57/4	646/3
Liver cirrhosis	-/+	6/55	102/547
Tumor number	1/>1	47/14	507/142
Tumor capsular	imcomplete/complete	19/42	258/391
TMD	cm	4.37±2.84	4.89±3.23
PBT	-/+	59/2	630/19
Differentiation	poor/moderate/well	0/46/15	13/538/98
MVI	-/+	42/19	446/203
AFP	ng/mL ± SD	1954.49±6558.55	3274.63±7588.34
AFPDAY		0.07±0.02	0.14±0.06
E50time	day	11.78±20.47	3.92±0.92
WBC	1 × 10^9^/L ± SD	5.94±2.95	5.62±2.62
PLT	1 × 10^9^/L ± SD	119.79±55.94	143.94±60.64
PA	g/L± SD	0.20±0.06	0.21±0.05
TB	umol /L ± SD	13.27±5.86	12.71±5.28
ALB	g/L± SD	40.38±2.86	40.70±3.31
ALT	U/L ± SD	68.89±186.11	43.92±77.62
GGT	U/L ± SD	79.62±58.49	75.25±73.93
ALP	U/L ± SD	87.85±32.99	82.77±33.79
CEA	ng/mL ± SD	2.78±1.59	2.56±2.09
CA19-9	U/mL ± SD	35.80±40.25	22.70±22.53

Values are presented as n (%) or mean ± standard deviation(SD).

Abbreviation: ALT: alanine transaminase, AFP: alpha fetoprotein, CEA: carcinoembryonic antigen, CA19 -9: carbohydrate antigen 19 -9, HBsAg: hepatitis B surface antigen, HCV: anti-hepatitis C virus, HLNE: Hilar lymph node enlargement, TMD: tumor maximum dimension, MVI: microvascular invasion, AFPDAY: daily decrease of post-operation/preoperative AFP, E50time:half-life of AFP, WBC: white blood cell, PLT: blood platelet, PA: prealbumin, TB: total bilirubin, ALB: albumin, GGT: gamma-glutamyltransferase, ALP: alkaline phosphatase, HBV DNA 0/3/4/5/6:*10^0/3/4/5/6^

### Identification of independent risk factors based on COX and logistic regression analysis

Univariate Cox proportional hazards regression for OS indicated that A09, HLNE, Tumor number, TMD, PBT, MVI, AFPDAY, WBC, PLT, PA, ALB, ALT, GGT, ALP, CEA were significant for OS, but preoperative AFP and stratified AFP had no influence on OS regression. Multivariate COX regression with those variables found significant difference only in A09 (HR:0.46, 95 % CI: 0.233-0.889, p =0.021), tumor number (HR: 2.02, 95 % CI: 1.279-3.199, p=0.003), TMD (HR: 1.10, 95 % CI: 1.035-1.177, p=0.003), AFPDAY (HR: 35.35, 95 % CI: 4.203-297.311, p=0.001), GGT(HR: 1.00, 95 % CI: 1.000-1.006, p=0.022), CEA (HR: 1.11, 95 % CI: 1.045-1.186, p=0.001) (Supplementary Table 3).

Univariate Cox proportional hazards regression for RFS found that significant variables were A09, HBsAg, HLNE, Tumor number, TMD, tumor capsular, PBT, MVI, AFP, PA, ALB, ALT, GGT, ALP, CEA, and CA19-9. Multivariate COX regression with the above variables found significance only in Tumor number (HR: 1.76, 95 % CI: 1.297-2.397, p<0.000), tumor capsular (HR: 0.64, 95 % CI: 0.482-0.860, p=0.008), CEA (HR: 1.07, 95 % CI: 1.013-1.135, p=0.015), and TMD (HR: 1.12, 95 % CI: 1.074-1.165, p<0.000). AFP was a significant factor in the univariate model but not in the multi-variate analysis. A09 did not show significance in RFS model (Supplementary Table 3).

### A09 as an independent prognostic factor for HCC

**Figure 1:**
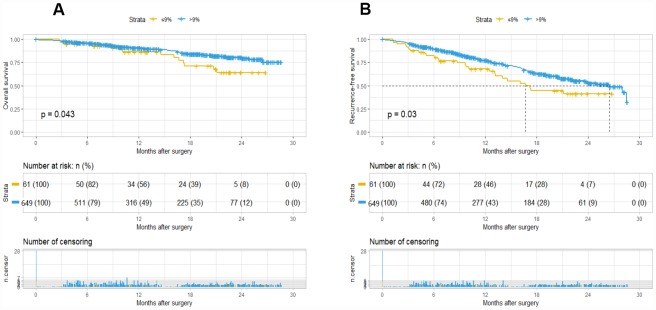
**Kaplan-Meier survival plot of OS and RFS based on A09.** The survival curve of overall survival (**A**) and recurrence-free survival (**B**).

The Kaplan-Meier curve showed that A09 was significantly different for OS (P=0.043) and RFS (P=0.03). A09>9% group had a better overall and recurrence-free survival. The median recurrence-free time was 17 months for A09≤9% group and 26 months for A09≥9% group, but median overall survival was not reached due to inadequate follow-up time (Figure 1). The logistic regression analysis revealed that A09 was associated with gender, AFP stratification, HLNE, PLT, and CA19-9. COX and logistic regression in combination did not found a confounding factor for A09, suggesting A09 as an independent prognostic factor for HCC (Supplementary Table 3). Furthermore, a comparison was made with the prognostic value of current international staging system in the HCC cohort. TNM 8^th^, BCLC, and liver function system (Child-Pugh, ALBI grade) developed a good stratification for overall and recurrence-free survival in the training cohort (Supplementary Figure 1, Supplementary Table 1–2). A09 were divided into substages, which showed a superior performance in TNM IB (RFS, p=0.038), BCBL B (RFS, p=0.093; OS, p=0.005), ALBI2 (RFS, p=0.021), Child-Pugh A (RFS, p=0.041), and Child-Pugh B (RFS/OS, p=0.00091) (Data not show).

### Incorporating A09 into TNM and BCLC staging system improves its predictive ability

To validate whether A09 is helpful in improving predictive ability, a nomogram was generated using the parameters selected through the multivariate COX regression. A09, tumor number, TMD, GGT, CEA (FDZS5) were incorporated into the model, and concordance of the nomogram at 0.72 showed a superior reliability to TNM (0.667) or BCLC (0.602). Tumor number, TMD and A09 (FDZS3) were incorporated into the nomogram after clinical selection. Concordance at a slightly lower level (0.687) also showed superiority to TNM and BCLC. A09 was further integrated into the TNM and BCLC staging systems to testify probable improvement in performance. A better concordance was observed in the training cohort after adding A09 into the incorporated staging system (0.672 VS 0.667 in TNM; 0.617 VS 0.602 in BCLC). When A09 was combined with individual assessing parameters (tumor number, tumor size, MVI, HLNE in TNM staging system; tumor number, tumor size, MVI, TB, and Child-Pugh in BCLC), a better concordance was observed than without A09 (0.683 VS 0.679 in TNM; 0.678 VS 0.671 in BCLC) (Table 2).

**Table 2:** Predictive concordance of FDZS, TNM and BCLC based on A09 or non-A09.

		**FDZS5**	**FDZS3**	**TNM**	**A09+TNM**	**BCLC**	**A09+BCLC**
		**Concordance**	**Concordance**	**Concordance**	**Concordance**	**Concordance**	**Concordance**
Training	Combined			0.667	0.672	0.602	0.617
	Separated	0.72	0.687	0.679	0.683	0.671	0.678
Internal validation	Combined			0.624	0.63	0.62	0.629
	Separated	0.63	0.608	0.641	0.649	0.653	0.664
External validation	Combined			0.788	0.85	0.688	0.85
	Separated	0.85	0.762	0.862	0.95	0.75	0.888

Calculated with tumor number, size, MVI, LN in TNM staging system and tumor number, size, MVI, TB, Child-Pugh in BCLC staging system(separated) or TNM, BCLC(combined).

**Figure 2:**
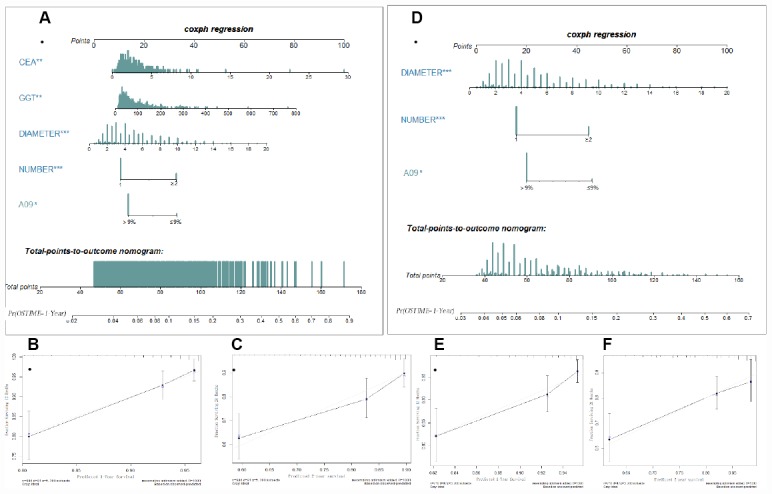
**Nomogram (FDZS5 and FDZS3) and validation to predict the probabilities of 1-year and 2-year overall survival for AFP elevated HCC patients after resection**. To use the FDZS5 (**A**) or FDZS3 (**D**), an individual patient’s value is located on each variable axis, and a line is drawn upward to determine the points for each variable. The sum of these points is located on the Total Points axis, and a line is drawn downward to the survival axes to determine the likelihood of 1-year OS. CEA, GGT, tumor diameter (cm), tumor number (1 or ≥2), A09 were used in the model. The calibration curve for predicting patient survival at (**B**, **E**) 1 years and (**C**, **F**) 2 years in the training set, Nomogram-predicted probability of overall survival is plotted on the x-axis; actual overall survival is plotted on the y-axis.

In the internal validation cohort, the same superiority was observed when A09 was incorporated into the staging system (in combination: 0.63 VS 0.624 in TNM; 0.629 VS 0.62 in BCLC; in separation: 0.649 VS 0.641 in TNM; 0.664 VS 0.653 in BCLC). In the external validation cohort, greater superiority was observed after incorporation of A09 (in combination: 0.85 VS 0.788 in TNM; 0.85 VS 0.688 in BCLC; in separation: 0.95 VS 0.862 in TNM; 0.888 VS 0.75 in BCLC). Notably, the external validation cohort showed the best reliability, an observation possibly attributable to the small sample size of it. Moreover, concordance was consistent with the training cohort in both internal (FDZS5:0.63; FDZS3:0.608) and external (FDZS5:0.85; FDZS3:0.762) validation cohorts, suggesting potential of A09 for the prognosis of AFP elevated HCC population (Figure 2).

## DISCUSSION

Predictive biomarkers should be better employed to help define the populations at risk of cancer burden. Such biomarkers constitute a valuable tool for screening the high-risk patients with HCC-related recurrences and deaths [5]. The main prognostic factors for HCC are tumor status, liver function and general tumor-related health status, and incorporation of those factors into such staging systems as TNM and the Barcelona-Clínic Liver Cancer (BCLC) [26]. These staging systems have been proposed to provide a clinical classification of HCC. However, few biomarkers have been incorporated into such systems due to inconsistent evidence. Other refined staging systems incorporated with serum biomarkers, such as CUPI, JIS and the Hong-Kong Liver Cancer (HKLC) staging system, and including, hinted the value of such biomarkers for classification.

However, biomarkers for accurate tumor prediction are yet to be found [4]. AFP, as an effective detection marker for early HCC, is confused for prognostic value of HCC. Although numerous studies have demonstrated that elevated AFP increases risk of tumor recurrence and survival after treatment, response to loco-regional therapies, risk of drop-out in patients on the waiting list for liver transplantation, and survival in advanced HCC [25, 27–30], the heterogeneity of the above studies prevents the formulation of a clear recommendation for using AFP in the prognosis of HCC [8]. Meanwhile, some studies reported its unreliability as a prognostic biomarker. Different viral infection and treatment may be a confounder for AFP prognostic assessment in HCC patients [22]. Insufficient data and strict rules for incorporating AFP as a prognostic or predictive marker into clinical practice limit its application.

Hence, we designed and conducted this two-center retrospective study to elucidate the association between perioperative serum AFP changes and prognosis for HCC patients after hepatectomy. This present study included 710 patients with preoperative AFP>40ng/ml and a decrease after hepatectomy. Interestingly, no significant differences were observed in preoperative AFP and AFP stratification (40–100/100–400/400/1000/>1000ng/ml), however, daily perioperative AFP decrease was significant to OS and RFS. To rectify the background of different preoperative AFP, it was divided by preoperative AFP, and X-tile was performed to determine a significant cut-off of 9% for the discrimination of prognosis, which was defined as A09. The at-risk population (A09>9%) showed a decrease of risk by 54% for OS and by 32% for RFS. The Kaplan-Meier curve also showed that A09 was significantly different for OS (P=0.043) and RFS (P=0.03), suggesting the independent prognostic value of perioperative decrease of AFP but not preoperative AFP, which was consistent with the previous study [19]. Considering abnormal elevation diagnostic value of AFP to HCC, it’s reasonable to understand that more quicker decrease ratio may present more thorough tumor clearance after surgery, and less possibility of recurrence.

Next, we wondered if there was an introduction value of A09 for HCC staging and liver function system. In prognostic staging system, significance was not observed except in TNM stage of IB and BCLC B. In the liver function system, ALBI 2 and Child-Pugh A-B were significantly different. Dividing the relatively small patient sample into sub-classes may result in the unsatisfactory significance. Considering the increased stratification ability after incorporating AFP into three Asian staging systems, we introduce such a perioperative detectable indicator into the current staging system to improve its accuracy. To validate this hypothesis, we incorporated A09 into TNM and BCLC staging system, calculated concordance in 3 cohorts, and found its superiority over the one without A09. The nomogram (FDZS5 and FDZS3) from the training cohort indicated that they had a better predictive ability than TNM and BCLC staging system, suggesting that perioperative AFP decrease could be a valuable parameter and that its incorporation could be able to improve the predictive ability of the staging system.

Furthermore, we observed that tumor number and maximum tumor dimension were significant factors for OS and RFS. Two other serum biomarkers, GGT and CEA, were also significant for OS, and tumor capsule and CEA for RFS. Meanwhile, we evaluated our cohort using the current system and verified a significant application of TNM 8^th^, BCLC, ALBI, and Child-Pugh system to classify the prognosis.

There are several limitations in this study. First, A09 is not applicable for the patients with normal AFP despite its superior discriminative ability for overall survival. Second, our study only recruited Chinese patients, and the results need verification in other regions other than China. Third, the exclusion of the patients advanced cancer sharped the overall assessment of staging system. Finally, unknown or unobserved confounding factors may contribute to potential bias because of the missing data.

## CONCLUSIONS

Decrease in perioperative AFP but not preoperative serum AFP is an independent factor for prognosis in HCC patients after hepatectomy. Cut-off A09 (daily decrease of AFP by 9%) significantly discriminate overall survival and recurrence-free survival and may be incorporated into TNM and BCLC staging systems to enhance staging classification for the population with elevated AFP. Furthermore, a predictive model was established for hepatectomy patients with AFP>40ng/ml, thus enable the clinical practitioners form a more precise clinical judgement.

## MATERIALS AND METHODS

### Participants and criteria

**Figure 3:**
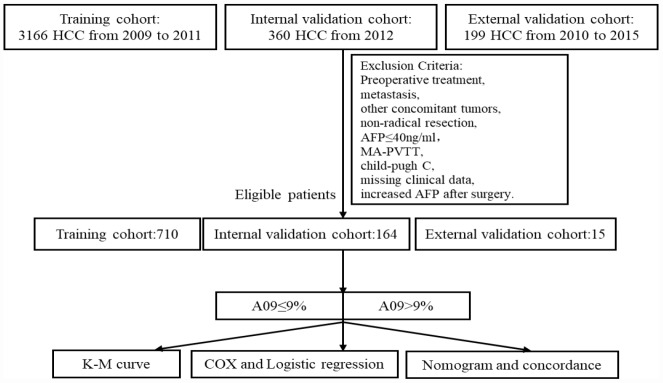
**Study flow chart.**

A total of 3526 HCC patients (aged 18 years or older) were enrolled in the study, who underwent resection at the Liver Cancer Institute of Zhongshan Hospital, Fudan University (FDZS) during 2009-2011 (3166 as training cohort) and 2012 (360 as internal validation cohort). An external validation cohort (200 patients) were collected from the Second Xiangya Hospital of Central South University (SXYCSU). All resections were performed or supervised by experienced hepatobiliary surgeons, and surgical specimens were histopathologically confirmed. Exclusion criteria were preoperative treatment, metastasis, other concomitant tumors, non-radical resection, AFP≤40ng/ml, macroscopic portal vein tumor thrombus (MA-PVTT), Child-Pugh C, missing clinical data, and increased AFP after surgery. Exclusion left 710 patients in the training cohort, 164 in the internal cohort, and 15 in the external cohort eligible for the study (Figure 3).

### Data source

The study with clinical data was approved and reviewed by the Ethics Committee of FDZS and SXYCSU. All data on the patients’ demographics, morbidity, postoperative mortality, and histological findings were obtained from the hospital medical system. All patients were followed up regularly at outpatient clinics of the two hospitals, tumor markers (AFP, CEA, CA19-9) and abdominal ultrasound were conducted every 3 months during the first 2 years, computed tomography or MRI were measured every 6 months or upon suspected recurrence, and further findings at the follow-ups were obtained via telephone by experienced researchers. The median follow-up time was 13.4 months (training), 46 months (internal) and 26 months (external), and the follow-up ended at January 2014 (training), December 2017 (internal), and April 2018 (external). The primary endpoint was death or the end of the follow-up, and the secondary endpoint was dropout from follow-up. Overall survival (OS) was defined as time from surgery until death from any cause, and recurrence-free survival (RFS) as the time from surgery until recurrence. A09 represented a daily post-operative decrease of AFP by 9% as compared to the preoperative level, the calculator was described as below.

$$\begin{array}{l} \text{A09}=\frac{\text{Pre-AFP}-\text{Post-AFP}}{\text{Period}\times \text{Pre-AFP}}\times \%\\ \begin{array}{l} \text{Pre-AFP\&nbsp;:\&nbsp;Latest\&nbsp;pre-operation\&nbsp;AFP}\\ \text{Post-AFP\&nbsp;:\&nbsp;Last\&nbsp;post-operation\&nbsp;AFP}\\ \text{Period\&nbsp;:\&nbsp;period\&nbsp;from\&nbsp;surgery\&nbsp;to\&nbsp;peri-operation\&nbsp;AFP\&nbsp;test} \end{array} \end{array}$$

Continuous variables are described as means with standard deviation, and categorical variables are presented as whole numbers and/or proportions as applicable. Two-sided p values of <0.05 were considered statistically significant. Statistical analyses were performed using SPSS 22 and R statistical software. Latest pre-operation examination (<15 days to operation) were set as baseline, the first post-operation AFP was test 1–3 days generally and the last peri-operation AFP value was regarded as the dominator (<10 days from operation). Parameters analyzed included age, gender, hepatitis B surface antigen (HBsAg), HBV DNA, anti-hepatitis C virus (HCV), Ascites, hilar lymph node enlargement (HLNE), liver cirrhosis, tumor capsular, tumor maximum dimension (TMD), perioperative blood transfusion (PBT), degree of differentiation, microvascular invasion (MVI), AFP, daily decrease of post-operation/preoperative AFP (AFPDAY), half-life of AFP (E50time), stratified AFP (40–100,100–400,400–1000, and >1000ng/ml), white blood cell (WBC), blood platelet (PLT), prealbumin (PA), total bilirubin (TB), albumin (ALB), alanine aminotransferase (ALT), gamma-glutamyl transferase (GGT), alkaline phosphatase (ALP), carcinoembryonic antigen (CEA), and carbohydrate antigen 19-9 (CA19-9). The regression models were established based on the Akaike’s information criterion. Univariate and multivariate Cox regressions were performed to assess variables listed above as potential determinants of survival. The survminer R packages were loaded into R version 3.5.1 to draw Kaplan-Meier curves of OS and RFS, and log-rank testing was employed for comparison between different variables. Variables with p<0.05 in univariate Cox regression were further evaluated using a variable selection procedure to identify independent prognostic factors. Only variables with a p-value less than 0.05 were retained in the final model. Hazard ratios (HR) and 95% confidence intervals (95% CI) were calculated for each variable. Univariate and multivariate logistic regression was performed to identify the confounding factors for A09, and only variables influencing A09 (logistic) and prognosis (COX) were defined as confounder [31, 32]. X-tile plots were used to determine cutoff point [33]. R packages (“rms”, “Hmisc”, “lattice”, “survival”, “Formula”, “ggplot2”, “foreign”, “regplot”) were loaded to draw the nomogram and calculate concordance [34]. TNM classification was based on the AJCC Cancer Staging Manual [8^th^ edition (2017) by springer New York, Inc.] [35]. Barcelona Clinic Liver Cancer (BCLC), ALBI grade [36] and Child-Pugh score were employed in the study.

### Ethics

The study with clinical data was approved by the Ethics Committee of the Zhongshan Hospital, Fudan University and Second Xiangya Hospital, Central South University. We clarify that all clinical data in this study was collected in patients who had given written informed consent.

## Supplementary Material

Supplementary Figure 1

Supplementary Tables

Supplementary Table 3
