# Supplementary material for: Daily decrease of post-operative alpha-fetoprotein by 9% discriminates prognosis of HCC: A multicenter retrospective study
Source: Aging (Albany NY). 2019 Dec 12;11(23):11111–23. doi: 10.18632/aging.102513 (PMC6932889; doi:10.18632/aging.102513)
Supplement: Supplementary Figure 1 [file aging-11-102513-s003..pdf]

SUPPLEMENTARY FIGURE

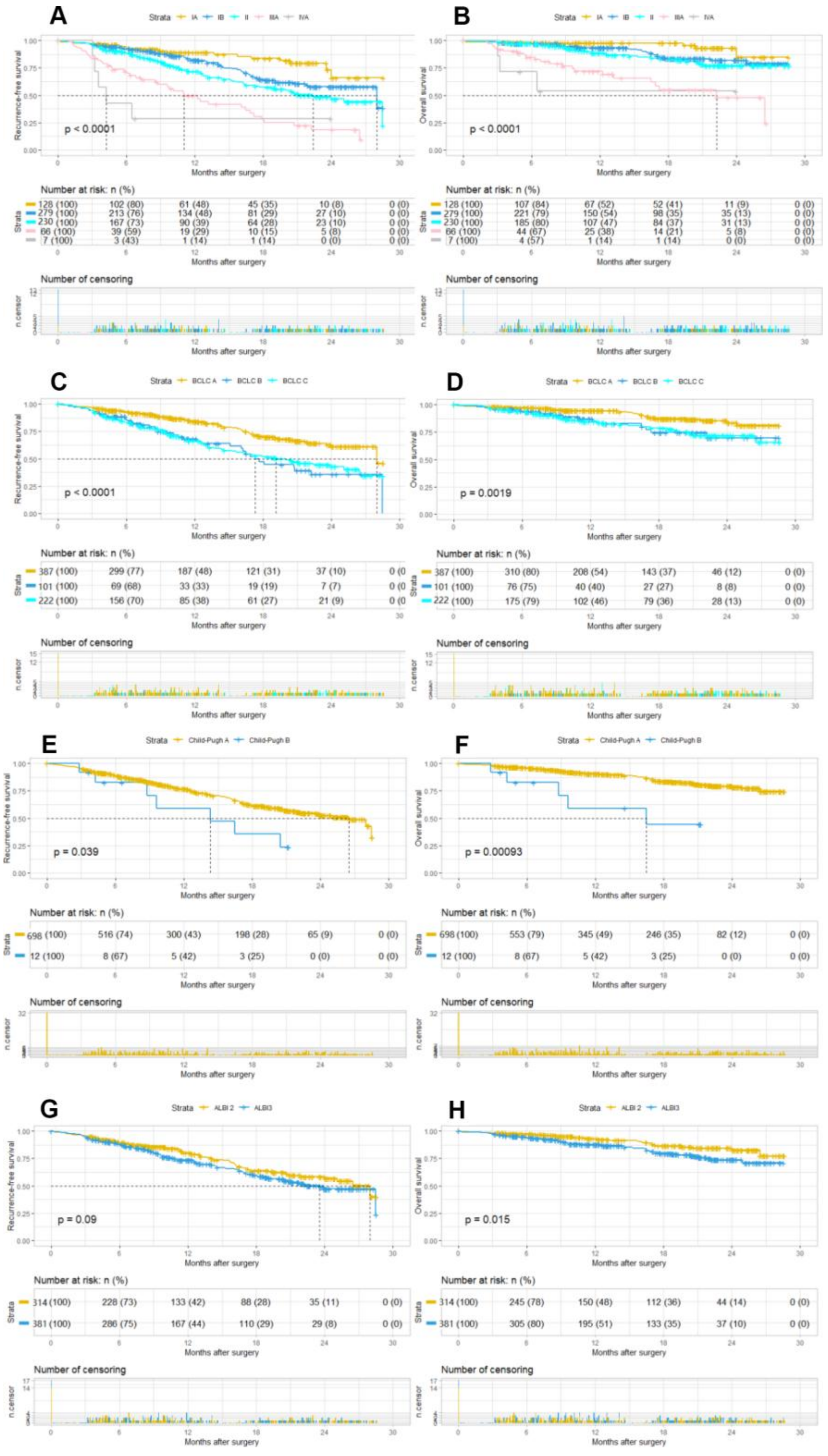

Supplementary Figure 1. K-M curves for TNM, BCLC, Child-Pugh and ALBI system in the training cohort. The survival curve of recurrence-free survival and overall survival based on TNM staging system (**A**, **B**), BCLC staging system (**C**, **D**), Child-Pugh score system (**E**, **F**), ALBI grade system (**G**, **H**).
