# Supplementary material for: Daily decrease of post-operative alpha-fetoprotein by 9% discriminates prognosis of HCC: A multicenter retrospective study
Source: Aging (Albany NY). 2019 Dec 12;11(23):11111–23. doi: 10.18632/aging.102513 (PMC6932889; doi:10.18632/aging.102513)
Supplement: Supplementary Tables [file aging-11-102513-s002..pdf]

SUPPLEMENTARY TABLES

Supplementary Table 1. Demographic of TNM, BCLC, ALBI, Child-Pugh system in the training cohort.

Characteristic	Variable	A09≤9%(n=61)	A09>9%(n=649)
TNM	IA/IB/II/IIIA/IVA	14/20/18/5/4	114/259/212/61/3
BCLC	A/B/C/	35/7/19	352/94/203
ALBI	1/2/3	0/24/37	0/290/344
Child-Pugh	A/B/C	60/1/0	638/11/0

Supplementary Table 2. Cox regression analyses (RFS/OS) based on TNM, BCLC, ALBI Child-Pugh system.

Characteristic	Variable	Overall survival		Recurrence-free survival	
		Univariate Cox regression		Univariate Cox regression	
		HR(95% CI)	P value[a]	HR(95% CI)	P value[a]
TNM	IA	reference	0.000	reference	0.000
	IB	2.39(0.996-5.749)		1.81(1.092-3.006)	
	II	3.01(1.254-7.208)		2.60(1.574-4.287)	
	IIIA	9.59(3.887-23.680)		6.01(3.479-10.390)	
	IVA	18.94(4.710-76.153)		9.67(3.601-25.981)	
BCLC	A	reference	0.003	reference	0.000
	B	2.06(1.137-3.717)		2.14(1.462-3.122)	
	C	2.12(1.350-3.331)		2.01(1.494-2.703)	
ALBI	2/3	1.71(1.102-2.638)	0.016	1.27(0.963-1.674)	0.091
Child-Pugh	A/B	4.09(1.655-10.082)	0.002	2.17(1.021-4.612)	0.044

Please browse Full Text version to see the data of Supplementary Table 3

Supplementary Table 3. Identification of independent risk factors based on COX and logistic regression analysis.
