# Supplementary material for: Daily decrease of post-operative alpha-fetoprotein by 9% discriminates prognosis of HCC: A multicenter retrospective study
Source: Aging (Albany NY). 2019 Dec 12;11(23):11111–23. doi: 10.18632/aging.102513 (PMC6932889; doi:10.18632/aging.102513)
Supplement: Supplementary Table 3 [file aging-11-102513-s001..docx]

Supplementary Table 3. Identification of independent risk factors based on COX and logistic regression analysis.
